# Editorial: Conduction system pacing: What’s missing for the paradigm shift?

**DOI:** 10.3389/fcvm.2023.1163370

**Published:** 2023-03-24

**Authors:** Lina Marcantoni, Francesco Zanon, Matteo Anselmino

**Affiliations:** ^1^ Arrhythmia and Electrophysiology Unit, Division of Cardiology, Santa Maria Della Misericordia General Hospital, Rovigo, Italy; ^2^ Cardiology Division, “Città della Salute e della Scienza di Torino” Hospital, Department of Medical Sciences, University of Turin, Turin, Italy

**Keywords:** arrhythimas, heart failiure, pacing (left bundle branch block), pacing & electrophysiology, conduction system

**Editorial on the Research Topic** Conduction system pacing: What’s missing for the paradigm shift?

Since 1950 pacemaker technology has experienced tremendous improvements, however, despite the evident and well-known clinical benefits, right ventricle apical pacing, being non-physiologic, may secondarily induce, in a not neglectable percentage of patients, undesired detrimental effects (1). Conduction System Pacing (CSP), namely His Bundle Pacing (HBP) and Left Bundle Branch Pacing (LBBP), has therefore gained increasing attention, and presents today the potential to become the first pacing modality in many clinical scenarios. Through the direct capture of the His-Purkinje system CSP maintains electrical and mechanical physiology in patients with narrow QRS, whereas potentially restores ventricular synchrony in case of underlying bundle branch blocks (2, 3).

In the early 2000s, the restricted number of available tools confined HBP in the hands of pioneers that could only share small, single-center experiences. Further knowledge on cardiac pacing physiology and development of new dedicated tools by the industry has, instead, favored the definitive CSP spread up (4). Three-dimensional sheaths equipped with septal curves facilitate the perpendicular lead orientation towards the septum, favoring lead fixation even in complex anatomies as those of patients with dilated heart or underlying structural disease. The availability of different designs and sizes ease the path to successful CSP not only by lumenless fixed screw, but also for stylet driven leads, adapting to the characteristics of any candidate. Non less importantly, the integration with electroanatomical mapping systems further facilitates the procedure by reducing learning curves and radiation exposure to the patient and the staff (5).

Through contributions from leading experts in the field, the present Special Issue presents a contemporary perspective on CSP. The increasing body of evidence surely confirms the more than promising outcomes of this innovative approach, however, by highlighting indistinctively both positive and negative insights, places emphasis on what is already clear and what, instead, is still lacking for routine CSP in clinical practice. Based on the original research, reviews, brief reports, and opinion papers included in the Issue two main considerations emerge.

The first is that research on LBBP outnumbers by far that on HBP. The likely explanation relates to the less technically challenging procedure compared to HBP, with low pacing threshold and appropriate sensing values more easily achieved. The limited experience with LBBB, compared to HBP, however requires further research and dedicated studies to fully uncover all underlying aspects and mechanisms. The reader of the Issue will find insights on the implant technique Pooter et al. and the in-depth electrophysiological features of the three different capture modes occurring during LBBP: selective, non-selective or left ventricular septal pacing Curila et al. Original aspects on LB trunk or LB fascicular capture are also described Liu et al.

The second consideration that emerges is that the general feeling of the Electrophysiology community is that CSP may represent a real alternative to standard biventricular pacing (BiV) for resynchronization purposes in heart failure patients that remain symptomatic despite optimal medical treatment Gui et al., Jiang et al., Hua et al., Fu et al. Heart failure CSP implants have been broadly performed, although they have yet to become a standard, guideline-recommended approach. Within clinical studies registered on ClinicalTrials.gov, about 30 in the recruitment phase relate to CSP, and, within these, the majority investigates this innovative pacing technique as an alternative to standard BiV by classical epicardial left ventricle lead placed through coronary sinus branching.

Overall, the present Issue supports the evidence that a true paradigm shift appears compelling. Before recommending CSP as first line treatment for both proximal and distal conduction disturbances and, even more, as an alternative to standard BiV, the Electrophysiology community, however, awaits larger experiences. Evidence from randomized trials is to date lacking, and urgently needed before recommending CSP as routine clinical practice. There is, however, no doubt that CSP will play (in fact, it already does) a central role in cardiac pacing strategies. Ongoing research and implementation of new dedicated devices and algorithms will permit to decide if CSP will become the default approach, enabling all Electrophysiologist to abandon right ventricle apical pacing, particularly in patients with expected high pacing burden. In the meantime, we hope the readers of Frontiers in Cardiovascular Medicine will find the current Special Issue helpful in broadening their understanding on CSP.
